# The impact of social media on adolescent energy drink consumption

**DOI:** 10.1097/MD.0000000000038041

**Published:** 2024-05-10

**Authors:** Nawal A. Alissa

**Affiliations:** a Department of Community Health Sciences, College of Applied Medical Sciences, King Saud University, Riyadh, Saudi Arabia.

**Keywords:** adolescents, energy drinks, social media

## Abstract

This study aimed to investigate the effects of social media on energy drink consumption among adolescents in Saudi Arabia. An online survey including demographic characteristics (3 questions), consumption patterns of energy drinks (5 questions), and Social Media Effects Scale (4 questions) was completed by 860 Saudi Arabian adolescents. Data were analyzed using Statistical Package for the Social Science version 29, using descriptive statistics and correlation to measure the relationship between social media and energy drink consumption. The results showed that nearly 82% of the adolescent respondents consumed energy drinks once to twice a week. Social media was the most common source of information on energy drinks (42.0%). The major findings of this study showed a positive correlation (*r* = .592, *P* > .05) between social media use and consumption of energy drinks. The study found that the average score for the Social Media Effects Scale was 5.75 out of 8, or 71.87%, indicating that social media influences the energy drinks consumption of roughly two-thirds of the study participants. Tailored action plans are required to raise awareness of the negative effects of energy drinks and change consumption patterns among the adolescent population due to a lack of knowledge and poorly controlled legislation on energy drinks.

## 1. Introduction

Energy drinks are sugar-sweetened beverages with over 32.4 mg of caffeine per 6 ounce.^[1]^ Energy drinks are a popular source of caffeine for adolescents and young adults.^[2]^ Marketers target this group with advertisements that focus on taste, color preferences, and risks.^[3]^ These drinks are popular among young consumers for a variety of reasons, such as meeting daily vitamin requirements, boosting energy levels, enhancing physical abilities, and aiding in weight loss.^[4]^

Energy drink consumption has become a worldwide problem, and it is particularly popular among adolescents, despite its negative effects on health, such as its effect on cardiovascular and cerebrovascular responses,^[1]^ due to its high caffeine and sugar content.^[5]^ Energy drinks help adolescents engage in other unhealthy habits that have negative effects on their health, including obesity and insomnia, a host of other health issues, and mental health issues.^[6]^ Previous research has found associations between energy drink consumption and late bedtime and an unhealthy diet.^[2]^

Several studies have reported high-energy drink consumption among adolescents. A study was conducted in the United States of America to examine trends in energy drink consumption from 2003 to 2016 using nutrition data from the National Health Examination Survey. Feeding on 9911 adolescents aged 12 to 19, 12,103 youths aged 20 to 39, and 11,245 adults aged 40 to 50. Logistic regression was used to calculate the percentage of energy drink consumers. Significant increases were observed across all age groups, with adolescent consumers rising from 0.2% to 1.4%, youth from 0.5% to 5.5%, and middle-aged adults from 0.0% to 1.2%.^[7]^ An Italian study was also conducted to find out trends in the consumption of energy drinks among school students during the period 2008 to 2019, using data from the European School Survey Project on Alcohol and Other Drugs-Italia2008 to ESPAD-Italia2019. The results showed that the prevalence of energy drink consumption increased significantly for males from 64.5% to 75.7%, and females from 46.8% to 61.8%.^[8]^

A study of 6902 adolescents in Germany from 2016 to 2018 found that 61.7% had been consuming energy drinks for their entire lives and 21.4% had started within the last 30 days.^[2]^ Similarly, a study in Finland found that the weekly consumption of energy drinks among Finnish adolescents increased from 18.2% in 2014 to 24.4% by 2018.^[9]^ Furthermore, a study conducted in Australia on adolescent students in 25 schools showed that 51.2% of 3688 participants consumed energy drinks, 23.4% reported that they consumed monthly, 19.2% consumed weekly, and 2% consumed energy drinks daily.^[10]^

Consumption of energy drinks has reached overwhelming proportions in many countries around the world, and Saudi Arabia is no exception. Energy drinks are widely available and accessible. Several studies have been conducted in Saudi Arabia, with the results indicating widespread use of energy drinks. It was reported that Hail City has the highest consumption rate, with a percentage of 60%. Jeddah led with 59.9%, followed by Medina with 52.2%, and Dammam came in at 45.63%. With a percentage of 37.8%, Qassim is the least commonly used in energy drinks.^[11]^

In 2015, a cross-sectional observational study was conducted, with 783 participants responding. The findings revealed that more than half of the participants drank energy drinks once to 3 times per month. And 46.7% of the participants responded that mass media is a reason for the widespread consumption of energy drinks.^[12]^ Additionally, a cross-sectional study was also conducted at Imam Abdulrahman bin Faisal University, with 1255 students participating. According to the study, 245 participants consumed energy drinks, with 26.1% doing so to keep friends company and 23.3% to stay awake.^[13]^ A recent study in Riyadh examined the prevalence of energy drink consumption among students at King Saud University and Imam Muhammad bin Saud University reported that 36.88% of students began drinking energy drinks in middle school, with 53.6% citing delicious taste as their primary reason for doing so.^[14]^

A variety of factors influence energy drinks consumption. According to some studies, excessive consumption of energy drinks is linked to a variety of factors, including peer pressure, smoking, a lack of knowledge about the ingredients, and living away from parents.^[13]^ However, data addressing social media as having a significant impact on energy drink consumption and new trends are scarce in Saudi Arabia. The promotion of unhealthy products through websites and social media has attracted the attention of healthcare professionals because of its potential impact on consumption behaviors.

Social media is an Internet-based platform where users can present themselves in real-time or asynchronously to both broad and narrow audiences who value user-generated content and interaction with others.^[15]^ The results of the Saudi Youth Development Survey were released by the General Authority for Statistics in 2019. According to the survey, approximately 67% of the Saudi population was aged 0 to 34, with the 15 to 34 age group accounting for nearly 37% of the total population (51.03% and 48.97% for males and females, respectively). According to the survey results, 98.43% had accounts on social networking sites.^[16]^ In Saudi Arabia, 68% of the population used social media in 2019. Saudi Arabia is among the world most active Twitter users.^[17]^ Adolescent energy drink consumption is being influenced by social media via advertisements, news, and posts on various platforms. Young adults are a high-risk group because they consume more unhealthy foods and beverages than other age groups, and they spend a significant amount of their time on the Internet.^[18]^

Energy drinks are marketed on social media platforms that are commonly viewed by adolescents, which may encourage youth to consume energy drinks.^[19]^ Energy drink marketing continues to exist in various forms. The study discovered energy drink marketing on the TikTok platform, which is popular among children and adolescents. There were 197 energy drink-related clips discovered, totaling over 70 million views. Children and adolescents accounted for 22% and 67% of all views. The clips show a positive attitude toward energy drinks, which may encourage adolescents to increase their consumption of energy drinks.^[20]^

According to the researchers’ best knowledge, no research has been published in Saudi Arabia that addresses the impact of social media on energy drink consumption among adolescents. Studies conducted in Saudi Arabia focused on the prevalence, factors, and reasons for consuming energy drinks among adolescents. The association between social media and energy drink consumption among adolescents has not been studied. Therefore, the purpose of this study was to investigate the effects of social media on energy drink consumption among adolescents in Saudi Arabia.

## 2. Methodology

### 2.1. Study design and study population

This study used a cross-sectional, correlational design to investigate how social media influences energy drink consumption among Saudi adolescents. The study was conducted in Saudi Arabia using an online survey. The survey, which was powered by Qualtrics software, was used to recruit the study participants. A survey link or Quick response code was sent via social media.

In order to take part in this study, the participants had to be between the ages of 12 and 18, lived in Saudi Arabia, and consume energy drinks at least once a month. Adolescents who reported never consumed or consumed less than once per month were excluded from the study. The respondents who said “never” or “less than once per month” of consuming energy drinks were thanked for their interest in this study but were told their current consuming behaviors do not match the participation criteria for the study. The data was collected between the months of June and July 2023.

### 2.2. Study sample

The study participants were recruited from a convenience sample of eligible individuals. Participants were recruited for this study via social media (WhatsApp, Twitter, Snapchat, and Instagram). The geographical region was taken into account in order to obtain a representative sample. The aim of the study was clearly explained at the beginning of the survey invitation letter. The online survey received responses from 1062 Saudi adolescents. The study excluded 202 participants with incomplete responses, leaving a sample size of 860 for analysis.

### 2.3. Study tool

This study adapted and utilized a previously validated questionnaire.^[21]^ To achieve the study aim, a short questionnaire (12 items) was distributed. It is comprised of 3 sections. The first section (3 items) asked the participants about their sociodemographic characteristics (age, gender, and place of residence). The second section (5 items) explored consumption patterns of energy drinks. For example, participants were asked to respond to the statement “source of information on energy drinks.” Participants were given the following choices: advertising, friends, the family, social media, other. The third section, Social Media Effects Scale, (4 items) explored the impact of social media on energy drink consumption. For example, participants were asked to respond to the statement “I believe that celebrities endorsing products and services on social media is interesting.” The item response options included a 3-point Likert scale ranging from 0 (Disagree) to 2 (Agree). Items were scored as Disagree = 0, Neutral = 1, Agree = 2. Higher composite scores on the overall scale indicated increased impact of social media on energy drink consumption.

The total possible score for this scale ranged from 0 to 8. The participants’ scores were interpreted using the midpoint of the scale highest possible score (equal to 4). The lower the score, the less likely the participant was affected by social media in consuming energy drinks.

### 2.4. Ethical considerations

This study was approved by the Research Ethics Committee of King Saud University. The participants were free to participate, and they were assured that their identities would not be revealed.

### 2.5. Data analysis

Data were analyzed using Statistical Package for Social Science software, version (29). Descriptive and inferential statistical data analyses were applied in the study with *P* < .05 considered significant. The independent variables in this study included demographic characteristics and pattern of energy drinks consumption. The dependent variable was the impact of social media on energy drinks consumption. Frequencies (n) and percentages (%) were run to determine pattern of energy drinks consumption and social media influences on energy drinks consumption. Quantitative data were analyzed by using the Pearson Correlation Coefficient and 1-sample *t* test to determine the relationship between energy drink consumption and social media.

## 3. Results

### 3.1. Demographic data of the study participants

The study examined the impact of social media on energy drinks of 860 Saudi adolescents. The majority of the participants (68.2%) were females, with 72.7% falling between the ages of 16 and 18 years. The study included participants from various regions of Saudi Arabia, but the majority were from the central region (74.0%) (see Table 1).

**Table 1:** Demographics characteristic among participants (n = 860).

Demographic group	Subcategory	Frequency
Gender	Female	587
	Male	273
Age	12–15	235
	16–18	625
Place of residence	Central	637
	West	87
	South	112
	East	24

### 3.2. Energy drinks consumption

Weekly consumption was measured based on frequency of consumption of energy drinks in separate question. Participants were asked, “How frequently did you consume energy drinks in the last week?” The answers varied from “none” to “Five times or more per week.” The majority of the participants (81.8%) reported consuming energy drinks once to twice a week. Only (18.2%) of participants reported consuming energy drinks 3 to 4 times a week. There were no responses for consuming energy drinks “five times or more per week.” Males reported higher weekly consumption than females (41% vs 30%, *P* < .001).

The Saudi adolescents’ source of information on energy drink consumption was measured. Social media was the most common source of information on energy drinks (42.0%), followed by friends (27.3%), and family (17.7). Advertisements were reported as the least common source of information on energy drinks (13.0%).

The reasons for consuming energy drinks were examined. The primary reasons for consuming energy drinks were taste and flavor (63.6%), share with friends (18.2%), gaining energy (13.2%), and to try them (5.0%), with significant gender differences (*P* < .001). The majority of adolescents (59.1%) identified energy drinks as soft drinks.

### 3.3. The impact of social media on energy drinks consumption

In response to the question “The most used social media application,” 40.9% of participants use TikTok application, while 31.8% use Snapchat application and 22.7% use Twitter application. Instagram application was the less used among Saudi adolescents (4.6%).

The vast majority of the study participants (67.5%) believed that celebrities endorsing products and services on social media are interesting, while 18.7% disagreed and 13.8% neutral.

For the statement “I believe that celebrities endorsing products and services on social media is very informative,” 47.8% of participants agreed, while 29.7% disagreed and 22.5% neutral.

In answer to the question “I believe that celebrities endorsing products and services on social media is reliable,” 54.5% disagreed, while 36.4% neutral and 9.1% of participants agreed.

Approximately half of the respondents 49.8% did not agree with the statement “When a celebrity announced the product, I became interested in purchasing it,” 27.3% neutral, and 22.7% of participants agreed.

Responses given by the participants in this study showed a positive correlation between social media and the consumption of energy drinks (*r* = .592). The correlation was not statistically significant, *P* value = .273 > .05.

Data from this study shows participants’ responses to items measuring the impact of social media on energy drink consumption. The question response options include a 3-point Likert scale ranging from 0 (Disagree) to 2 (Agree). The questions were scored as Disagree = 0, Neutral = 1, Agree = 2. Item scores were summed for the total scale score. Higher scores on the overall scale indicate increased the impact of social media on energy drink consumption. The total score of 4 items is 8 points with 4 points as the average score. After analysis of participants’ responses, the mean total score on the overall scale was 5.75 out of 8 (SD = 0.92). The impact of social media on energy drink consumption was above the average score. A 1-sample *t* test suggested the obtained mean of 5.75 is not significantly higher than the average 4 (*P* > .05).

## 4. Discussion

This study used a cross-sectional online survey to examine the effects of social media on energy drink consumption. The surveys were examined, and data were analyzed using descriptive statistics, measures of central tendency, frequencies, Pearson Correlation, and 1-sample *t* test to investigate the impact of social media on energy drink consumption. Majority of participant were aged 16 to 18 and more than half of the respondents were females.

The major findings of this study showed a positive correlation (*r* = .592, *P* > .05) between social media use and consumption of energy drinks, suggesting that the have higher use of social media by adolescents has a greater influence on their energy drink consumption. This result concurs with findings reported by a previous study indicating a positive relationship between adolescent energy drink initiation and frequent exposure to energy drink advertisements.^[2]^ Canadian and Australian studies revealed that energy drink marketing is popular on digital platforms such as Twitch, Facebook gaming, and YouTube gaming that target young people, and those studies positively associated energy drink digital marketing with energy drink consumption.^[22]^ To counteract the effects of digital marketing through social media platforms on adolescent health, researchers have proposed regulating all forms of marketing, including online marketing.^[18]^

The study found that the average score for the Social Media Effects Scale was 5.75 out of 8, or 71.87%, indicating that social media influences the energy drinks consumption of roughly two-thirds of the study participants. In a previous study in Taiwan, researchers found higher levels of efficacy for energy drink advertising on energy drinks consumption in seventh grade.^[23]^ A study conducted in the United States (US) on a large cohort of grade 8 and 11 students reported that adolescents who used media for longer hours had a higher odds of unhealthy food.^[24]^ However, because no previous research had been undertaken in the Middle East, the study findings were not compared to data from the region.

This study shows a disturbing situation in the consumption of energy drinks among adolescents in Saudi Arabia. Most participants (81.8%) reported consuming energy drinks once or twice a week. These findings are consistent with previous research conducted in various nations.^[2,23,25,26]^ These worrying statistics on the widespread consumption of energy drinks are concerning since the younger generation is still developing physically and psychologically and is likely not aware of the potential risks associated with this use. This study also showed that energy drinks were viewed as soft beverages by the majority of participants (59.1%), suggesting that a lack of knowledge concerning energy drinks was prominent among the participants.

In this study, males were shown to consume more energy drinks than females. This result also concurs with previous research indicating that in Saudi Arabia, more males than females reported weekly consumption.^[13]^ Also, these findings are consistent with other study indicating that male secondary school students showed a high usage of energy drinks.^[27]^

Observing the causes for the consumption of energy drinks, survey participants showed that they usually use energy drinks for taste and flavor. This result concurs with findings reported by a previous study that taste and flavor were the most important reasons for consuming energy drinks.^[21]^ However, this finding is not consistent with other studies reporting that the most common reason to use energy drinks was to stay awake.^[28]^

This study contributes to the current literature by confirming the prior worldwide trend of energy drinks consumption, as well as the potential negative impacts on health behavior. Because of the lack of knowledge and poorly controlled legislation of energy drinks, tailored action programs are needed to promote awareness of their adverse effects and modify consumption patterns among the adolescent population. The findings of this study emphasized the need for health promotion and health education actions aimed at adolescents’ energy drink consumption, with the goal of supporting adolescents’ abilities to maintain healthy daily rhythms and thus sustain their vitality. Health authorities and healthcare professionals should expand their awareness campaigns to educate adolescents about the negative effects of energy drinks. More rigorous evaluation is needed for the marketing of energy drinks on platforms popular with adolescents. Furthermore, the Saudi government should reconsider its regulations regarding the advertising of beverages on social media.

### 4.1. Strengths and limitations

The strengths of the study consist of a sizable sample that is nationally representative, measures that have been validated, and a range of indicators that cover the consumption patterns of energy drinks and social media effects on adolescents. However, this study has some limitations. The participants were asked to recall how frequently they have consumed energy drinks during a specific period. Thus, participants would not be able to precisely remember how frequently they have consumed energy drinks over that period of time. In addition, it is possible that the participants’ responses will change when they know their intake will be measured. The variables of interest were measured by self-report, and therefore, subject to bias, underreporting and overreporting. The study used a quantitative correlational design. Correlation does not imply causation, and correlational studies cannot prove causation. The study referred to adolescents’ population in Saudi Arabia. The results possibly will not be generalizable to other countries.

## 5. Conclusions

This study examined the effects of social media on energy drink consumption among adolescents in Saudi Arabia. This study indicated that the consumption of energy drinks was prevalent among male participants. This study found that social media platforms may have a negative impact on energy drinks consumption. Future studies to examine factors associated with the negative effect of social media on energy drinks consumption are warranted. Further, recommendations for imposing stricter regulations on the marketing of energy drinks from the regulatory authorities such as the Food and Drug Authority. Imposing regulations from the Ministry of Commerce to prevent the sale of energy drinks to those under the legal age. Raising awareness of the negative effects of excessive consumption of energy drinks with the help of government agencies such as the Ministry of Health and the Ministry of Education. Increasing parents’ awareness of the damage resulting from the misuse of social media sites and their impact on their children behavior by conducting awareness campaigns.

## Acknowledgments

The author would like to express her gratitude to all adolescents who participate in the research. Your open and sincere feedback helped us to develop an important understanding that will help shape a healthier future for our nation.

## Author contributions

**Conceptualization:** Nawal A. Alissa.

**Data curation:** Nawal A. Alissa.

**Formal analysis:** Nawal A. Alissa.

**Methodology:** Nawal A. Alissa.

**Resources:** Nawal A. Alissa.

**Supervision:** Nawal A. Alissa.

**Writing – review & editing:** Nawal A. Alissa.
